# Seasonal Patterns of Buruli Ulcer Incidence, Central Africa, 2002–2012

**DOI:** 10.3201/eid2108.141336

**Published:** 2015-08

**Authors:** Jordi Landier, Guillaume Constantin de Magny, Andres Garchitorena, Jean-François Guégan, Jean Gaudart, Laurent Marsollier, Philippe Le Gall, Tamara Giles-Vernick, Sara Eyangoh, Arnaud Fontanet, Gaëtan Texier

**Affiliations:** Institut Pasteur, Paris, France (J. Landier, T. Giles-Vernick, A. Fontanet);; Centre Pasteur du Cameroun, Réseau International des Instituts Pasteur, Yaoundé, Cameroon (J. Landier, S. Eyangoh, G. Texier);; UMR MIVEGEC, Montpellier, France (G. Constantin de Magny, A. Garchitorena, J.-F. Guégan);; Ecole des Hautes Etudes en Santé Publique, Rennes, France (A. Garchitorena, J.-F. Guégan);; UMR912-SESSTIM, Marseille, France (J. Gaudart, G. Texier);; INSERM and CHU et Université d’Angers, Angers, France (L. Marsollier);; Institut de Recherche pour le Développement, Gif sur Yvette, France (P. Le Gall);; Université Paris-Sud 11, Orsay, France. (P. Le Gall);; Chaire Santé et Développement, Conservatoire National des Arts et Métiers, Paris (A. Fontanet)

**Keywords:** Buruli ulcer, Mycobacterium ulcerans, wavelet analysis, seasonal variations, Cameroon, Central Africa, bacteria, tuberculosis and other mycobacteria

## Abstract

To determine when risk for Buruli ulcer is highest, we examined seasonal patterns in a highly disease-endemic area of Cameroon during 2002–2012. Cases peaked in March, suggesting that risk is highest during the high rainy season. During and after this season, populations should increase protective behaviors, and case detection efforts should be intensified.

Buruli ulcer (BU) is a severe infection caused by *Mycobacterium ulcerans*. Most affected are rural populations living in tropical areas with abundant wetlands (*1*). BU causes extensive, damaging skin lesions and often results in severe disabilities. Of the 4,000 cases reported to the World Health Organization by 14 countries in 2011, >95% originated in African countries around the Gulf of Guinea (*2*).

Much remains unknown about the mode of *M. ulcerans* transmission and the epidemiology of BU (*1*). Specifically, although the spatial distribution of BU in several settings has been addressed (*3*,*4*), most studies have examined only temporal variations of BU incidence in terms of yearly trends (*5*). Several observational studies have reported seasonal changes in the monthly number of cases and have hypothesized that cases are linked with rainfall variation (*6*–*8*). One spatiotemporal study in Australia showed that BU incidence was associated with rainfall variability with a 5-month lag and with total rainfall with a 19-month lag (*4*). However, none of these studies provided quantitative evidence of seasonal changes in BU incidence and their relationship with seasonal environmental changes. Indeed, a formal demonstration of such evidence requires a sufficiently long time series, large numbers of cases from a defined source population, and use of signal analysis techniques adapted to the constraints of BU disease surveillance and environmental data (*9*) (Technical Appendix). Therefore, we investigated the seasonality of BU case incidence during 2002–2012 in Akonolinga District, located in the highly BU-endemic region of the Nyong River valley in Centre Region, Cameroon.

## The Study

Relying on previous spatial analysis of BU incidence in Akonolinga District, we analyzed a series of cases that occurred in the highest BU-risk area of the district, located along the Nyong River upstream of Akonolinga (*3*). This area includes 24,469 inhabitants of the town of Akonolinga and 24 surrounding villages. We analyzed 562 new cases of BU that originated in this area from January 2002 through May 2012, after aggregation by month of diagnosis. Biological confirmation was obtained from the National Reference Centre for Mycobacteria for 354 (63%) cases. The BU incidence rate remained stable over the 10-year period at 2.2 cases/1,000 person-years.

**Figure 1:**
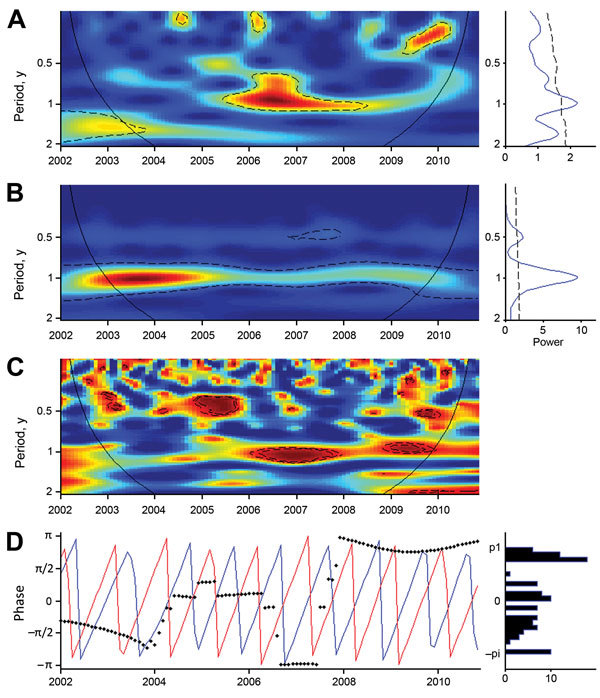
Wavelet analysis of Buruli ulcer (BU) case series and Nyong River flow, January 2002–December 2010. A, B) The color gradient indicates how well the wavelet of a given period adjusted with the series (power). The detection of periodic signals was performed within a confidence cone, which excluded the beginning and the end of the series where edge effects would be too likely (black solid line). Statistically significant zones are circled with dashed lines, indicating detection of significant periodic signals during the corresponding years. A) Wavelet power spectrum for the time series of BU cases: a seasonal signal with a 1-year period was detected from 2005 to 2011 (green to black), and this period was statistically significant from mid-2005 to the beginning of 2009 (dashed contour lines). B) Wavelet power spectrum for the Nyong River flow: the Nyong River flow series exhibits a statistically significant 1-year periodic signal during the whole period. C) Wavelet association between BU incidence and the Nyong River flow signal. The color gradient translates the association between the 2 signals. The dashed lines indicate statistically significant association, and the black line the confidence cone. D) Phase analysis for the 1-year period (expressed in multiples of π); BU cases are represented in blue and Nyong River flow variables in red.

Median BU incidence peaked in March, and a second peak occurred in September (Technical Appendix Figure 2), but monthly medians did not differ significantly (Kruskal-Wallis test, p = 0.149). Given the specificities (nonstationarity) of the BU case series, wavelet analysis was the appropriate method for analysis (Technical Appendix). A 1-year periodic signal was identified in the BU-case time series from 2005 to 2011, and this periodicity was statistically significant from mid-2005 to the beginning of 2009 (Figure 1).

Next, we analyzed the links between BU and seasonal changes by using wavelet association and phase analyses between BU case incidence and total monthly rainfall (in mm) or mean Nyong River flow (in cubic meters per second). Strong seasonality was found in the series of monthly total rainfall and of monthly mean Nyong River flow; a 1-year period and a weaker 6-month period corresponded to the 2 rainy seasons separated by a period of lesser rainfall (the small dry season, mid-July to mid-August) (Figure 1; Technical Appendix Figure 3). Because of its shape, the wavelet detected yearly rainfall oscillations between a minimum in December (dry season) and a maximum in July (the middle of the rainy period) instead of the maximal rainfall months of October and November (Technical Appendix Figure 3).

We assessed the association of the incident case signal with environmental variables (Technical Appendix). The 1-year periodic signal of the BU case series was associated with Nyong River flow from the end of 2005 to the end of 2009 (Figure 1) and with rainfall from the end of 2005 to the beginning of 2011 (Technical Appendix Figure 3). Under the assumption that changes in the environment preceded changes in BU incidence, phase analysis indicated that cases lagged 6 months behind Nyong River flow oscillations (Figure 1). When the 2 signals were associated, a 9-month lag behind rainfall oscillations was observed (Technical Appendix Figure 3).

## Conclusions

**Figure 2:**
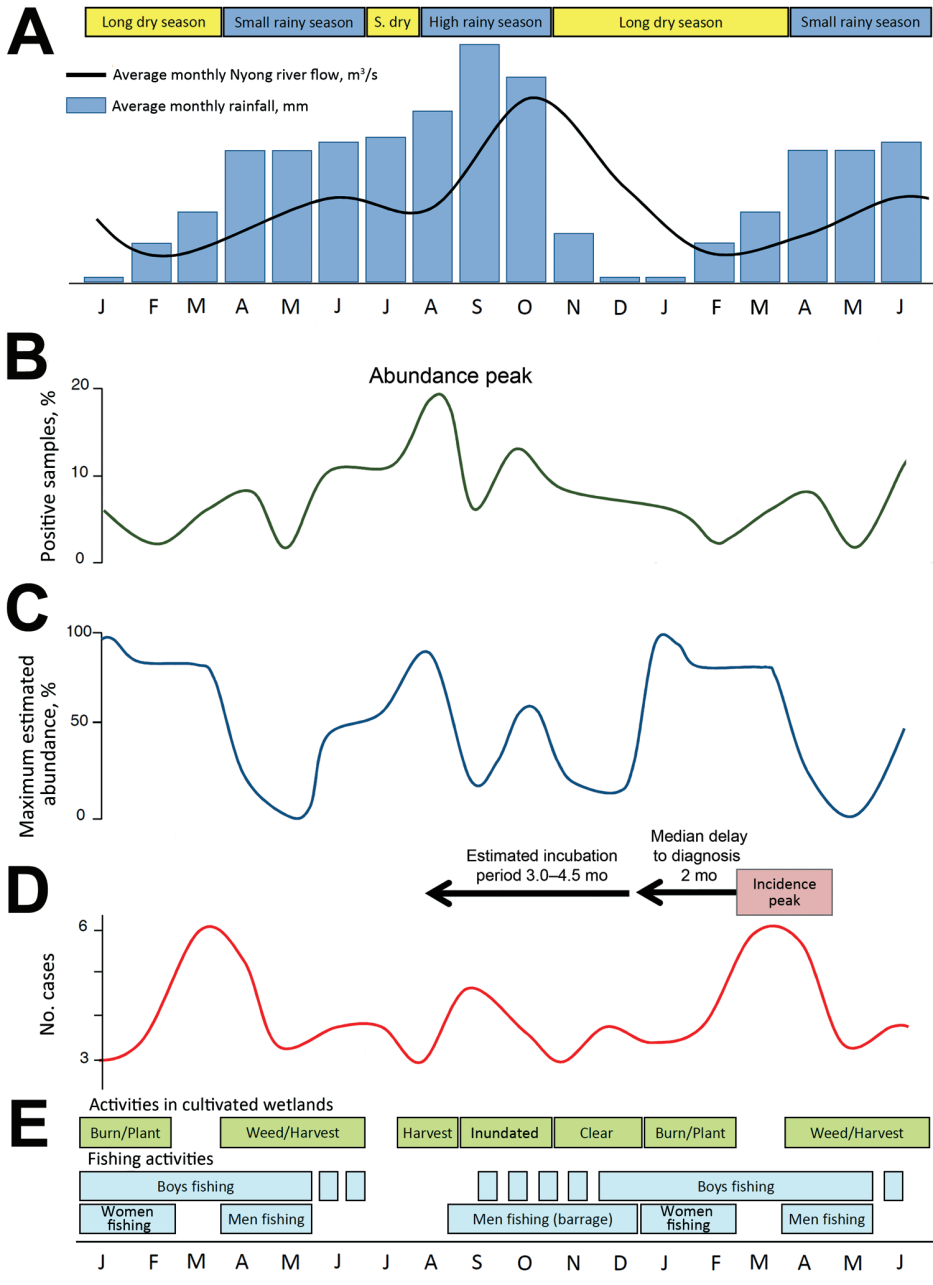
Schematic representation of the seasonal changes and possible links between the environment, *Mycobacterium ulcerans* presence, human exposure, and Buruli ulcer (BU) incidence in the Akonolinga district and the Nyong River valley, Cameroon, 2002–2012. For better visualization of delays, 18 months are shown. A) Average monthly rainfall and mean Nyong River flow (Technical Appendix). S, dry, short dry season. B) *M. ulcerans* prevalencein the aquatic environment (percentage of *M. ulcerans–*positive samples) (*14*). C) Estimated abundance of *M. ulcerans*–positive hemipterans (expressed as % of maximum abundance) (*14*). D) Monthly median number of BU cases detected in the Akonolinga district, 2002–2012 (this study). E) Selected activities involving contacts with environments in which risk for BU is high (T. Giles-Vernick, pers. comm., 2015).

In the BU-endemic focus of Akonolinga, Cameroon, significant 1-year seasonal variations in BU incidence occur. The incubation period for BU has been estimated to be ≈4.5 months when data from Australia are used (*10*) and ≈3 months when data from Uganda are used (*7*). The median delay between symptom onset and health care seeking was reported to be 5 weeks in Akonolinga (interquartile range 3–12 weeks), yielding a delay between infection and diagnosis of 5–6 months. Given this delay and a finding of BU diagnosis peaks during March–April, the number of infections would therefore be highest from August through October (Figure 2). Such a pattern was observed in the 1970s in Uganda (*6*,*7*) and Cameroon (*11*) and more recently in Côte d’Ivoire (*5*). In low BU-endemicity French Guiana, an overseas territory located near Brazil at the same latitude as Cameroon, periodic peaks after the 2 rainy seasons have been reported (*12*).

Variations in BU incidence result from variations in population exposure (*13*) combined with variations in environmental presence of *M. ulcerans* (*14*). We hypothesize that one of the main drivers of these variations is the seasonal flooding of the Nyong River, which rises 3–5 m from April through November, creating temporary bodies of water and swamps on a vast surface, deeply affecting the ecosystem.

Although *M*. *ulcerans* was identified year-round in specific environments such as permanent swamps, its presence and abundance were maximal during the rainy months, July–October (*14*) (Figure 2). Prevalence of *M. ulcerans* in rivers was high at the beginning of the rainy season and was high in flooded areas during the following small dry season and high rainy season (*14*).

Human activity patterns follow these seasonal changes, resulting in seasonal variations in exposure to *M. ulcerans*. In Uganda, the contribution of permanent swamps to BU risk and the increased risk associated with temporary swamps during the rainy season have been documented (*8*). According to residence, age, and/or sex, the inhabitants of the Akonolinga district face varying exposures to aquatic environments; during the period identified as high risk, populations frequent seasonally flooded environments for water collection, fishing, and harvest of dry season cultures (Figure 2) (*15*).

During the study period, the intensity of the association between BU incidence and rainfall or Nyong River flow varied. The seasonal signal was detected over 5 consecutive years and was strongest when yearly variations in the Nyong River flow were lower (2005, 2006, 2008), which could indicate transient forcing of BU incidence by seasonal phenomena. Assessment of the effects of lower frequency climatic events, such as El Niño Southern Oscillation, is needed. In French Guiana, where BU endemicity is low, such events were shown to affect BU incidence dynamics (*12*).

We showed that BU incidence in this region varies significantly by season and linked these variations to the fluctuations of *M. ulcerans* occurrence in the environment, which are probably driven by the dynamics of freshwater ecosystems of the Nyong River. In Akonolinga, during the high rainy season when risk for *M. ulcerans* transmission seems to be highest, populations should increase their protective behaviors, and case detection efforts should be intensified in subsequent months to ensure early diagnosis and access to care.

**Technical Appendix.** Additional materials, methods, and results for analysis of seasonal patterns of Buruli ulcer incidence, central Africa, 2002–2012.
