# Supplementary material for: Seasonal Patterns of Buruli Ulcer Incidence, Central Africa, 2002–2012
Source: Emerg Infect Dis. 2015 Aug;21(8):1414–7. doi: 10.3201/eid2108.141336 (PMC4517715; doi:10.3201/eid2108.141336)
Supplement: Supplementary file 1 — Technical Appendix. Additional materials, methods, and results for analysis of seasonal patterns of Buruli ulcer incidence, central Africa, 2002–2012. [file 14-1336-Techapp-s1.pdf]

Article DOI: http://dx.doi.org/10.3201/eid2108.141336

Seasonal Patterns of Buruli Ulcer Incidence, Central Africa, 2002–2012

Technical Appendix

Supplemental Material and Methods

Cases

This work was a registry-based study of the monthly number of Buruli ulcer cases who received a clinical diagnosis and treatment in Akonolinga Hospital, Centre Cameroon, from January 2002 to May 2012, as previously reported (*1*). A BU case was defined as a patient receiving a BU clinical diagnosis from a trained medical personnel in Akonolinga Hospital, which proved a reliable method in endemic areas (*2*). A confirmed case was defined as a patient for which laboratory identification of MU was obtained. Biologic confirmation of *M. ulcerans* infection was provided by the National Reference Centre for Mycobacteria in the Centre Pasteur du Cameroun in Yaoundé according to WHO guidelines (*3*). Detection of *M. ulcerans* (MU) was performed by microscopy, culture and PCR as previously described (*4*). PCR targeting MU-IS2404 has been available at the Centre Pasteur du Cameroun since 2001, and quantitative PCR was set up as early as 2009.

We restricted our analysis of BU incidence to villages located in the high BU-risk area previously identified (*1*). This high risk area was located along the Nyong River upstream and downstream from Akonolinga town (Technical Appendix Figure 1).

All incident new BU cases were included and aggregated by month of diagnosis at Akonolinga Hospital.

Environmental data

Because of a suspected link with water, water-related environmental variables included in the analysis. We used monthly total rainfall obtained from validated remote-sensing data (MARS project) which combines satellite data and rainfall measurements (*5*). We extracted the data from the 25x25km cell that overlapped with our study area. We also used the monthly average of Nyong river flow measured in the hydrologic station of

Mbalmayo, a city located ≈50 km downstream from Akonolinga area. This data was only available from January 2002 to December 2010 (*6*).

Ethics Statement

This study used anonymised case data, aggregated by village and by month, which were collected by the Service de Mycobactériologie of the Centre Pasteur du Cameroun as part of the surveillance activity of the National Reference Laboratory for BU in Cameroon, within the National BU Control Program. In this study, no intervention was performed (either diagnostic or therapeutic) and we only relied on a retrospective collection of anonymous cases authorized by the Cameroonian Ministry of Health.

Statistical Methods

Wavelet analysis was performed on the time-series of monthly BU cases to determine the significant oscillating modes. This method has been validated in epidemiology and was employed because epidemiologic and environmental time-series are typically nonstationary, i.e., their dominant periodic components change over time (*7–10*). These characteristics may render traditional spectral techniques, such as Fourier analysis, inappropriate to analyze the temporal trend of local variations in frequency and periodicity (*7*). We selected the Morlet wavelet for the wavelet decomposition in the periodic band between 0.3 (4 months) and 2 years over the 125 months duration (January 2002 to May 2012). Detection of periodic signals is performed within a confidence cone, which excludes the beginning and the end of the series where edge effects would be too likely (*7*). Statistical significance of the signal was computed on 1,000 simple bootstrapped series and the α = 5% significance level zones are circled with black dashed lines (*8*). The right panel of the wavelet spectrum corresponds to the global wavelet spectrum (black line) with its significant threshold value of 5% represented by the black dashed line (*8*).

Wavelet coherency was then used to identify and quantify possible statistical associations between two time series, e.g., the BU case time series and one of the environmental variables. Coherency is roughly similar to a classical correlation, but pertains to oscillating components in a given frequency mode for a given time period (*7*). Statistical significance of coherency was computed on 1,000 simple bootstrapped series and the α = 10% and α = 5% significance level zones are circled with black dashed lines. We also analyzed the phases in the time-series which enabled us to obtain information about the possible delay between incidence and environmental variables (i.e., in phase or out of phase relations). Phase analysis was complemented with the computation of the evolution of periodic components in

the 1-year mode. The phase difference between the two oscillating components (ΔT) was represented by the black dotted line. The right panel of the phase analysis corresponds to the phase difference histogram over the whole time period (black line). The phase difference can be converted into months (by multiplying by 12/2π): when investigating oscillations with a 12-month period, a phase difference of π corresponds to a 6-month time delay. Statistical analyses were performed using Matlab (version R2013a, The MathWorks, Naticks, Massachusetts, United States).

Supplementary Results

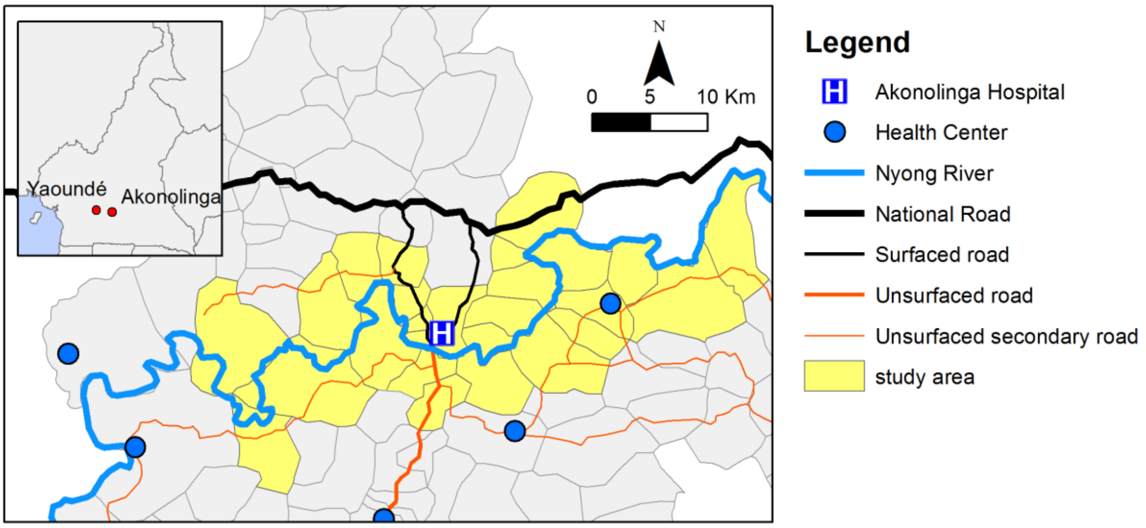

Technical Appendix Figure 1. Presentation of the study area displaying the location of Akonolinga Hospital, of the Health Centers and of the main roads. The boundaries of each village are displayed in dark gray.

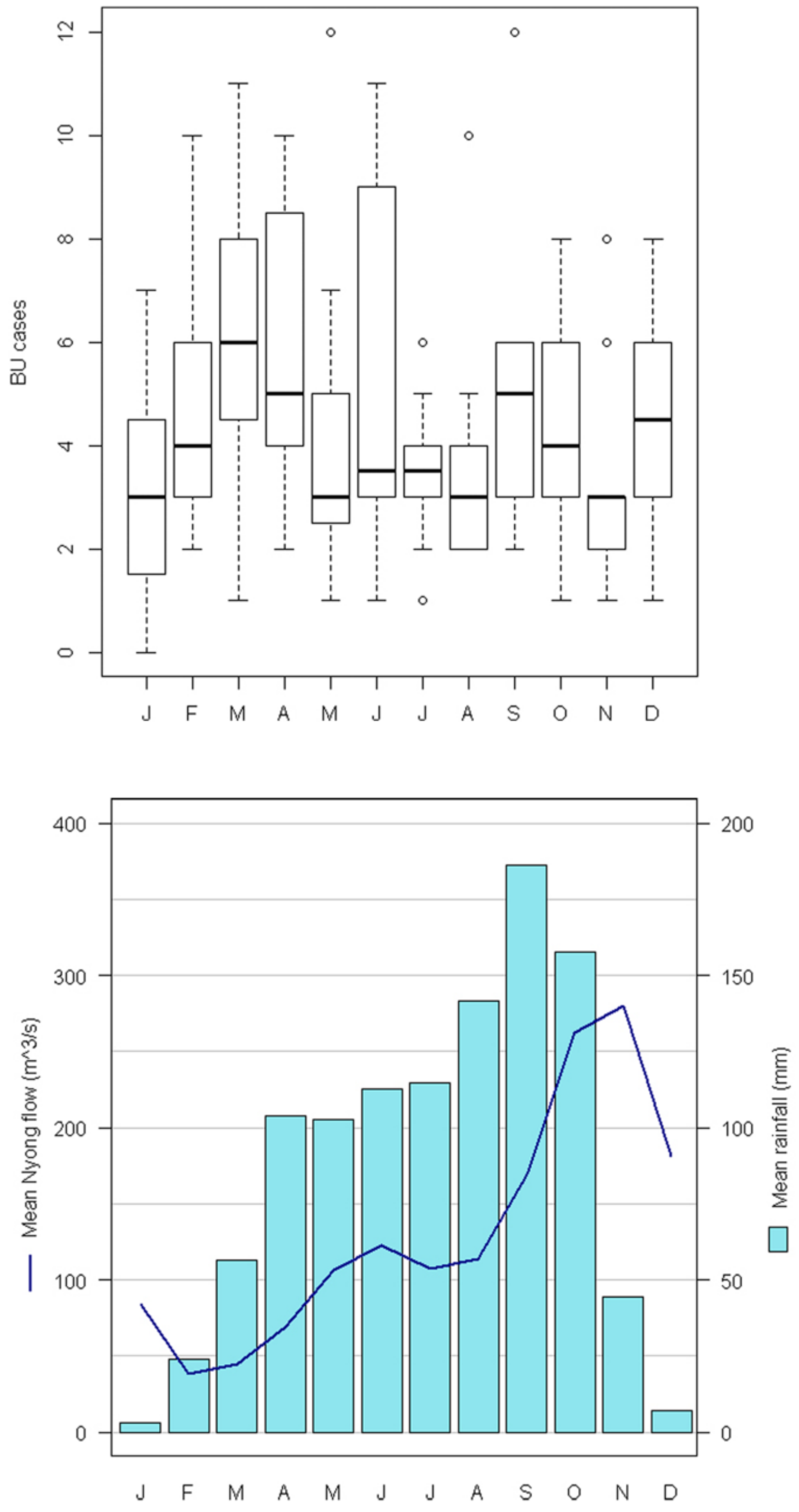

Technical Appendix Figure 2. Boxplot of monthly BU incidence data over the study period, and mean monthly rainfall and Nyong River flow.

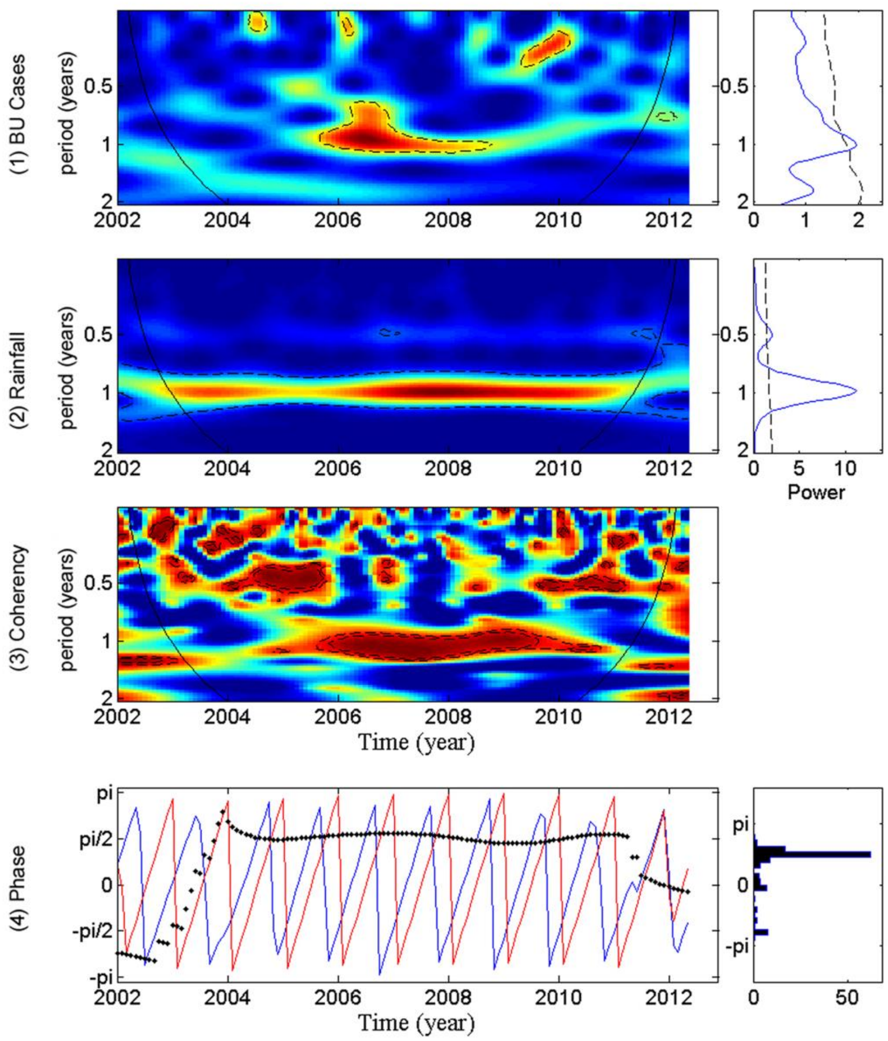

Technical Appendix Figure 3. Wavelet analysis of BU incidence and rainfall.

Panel 1: analysis for the time series of BU cases,

Panel 2: Nyong River flow

Panel 3: coherency between the two signals,

Panel 4: phase analysis for the 1-year period, BU cases are represented in blue and nyong flow variables in red.

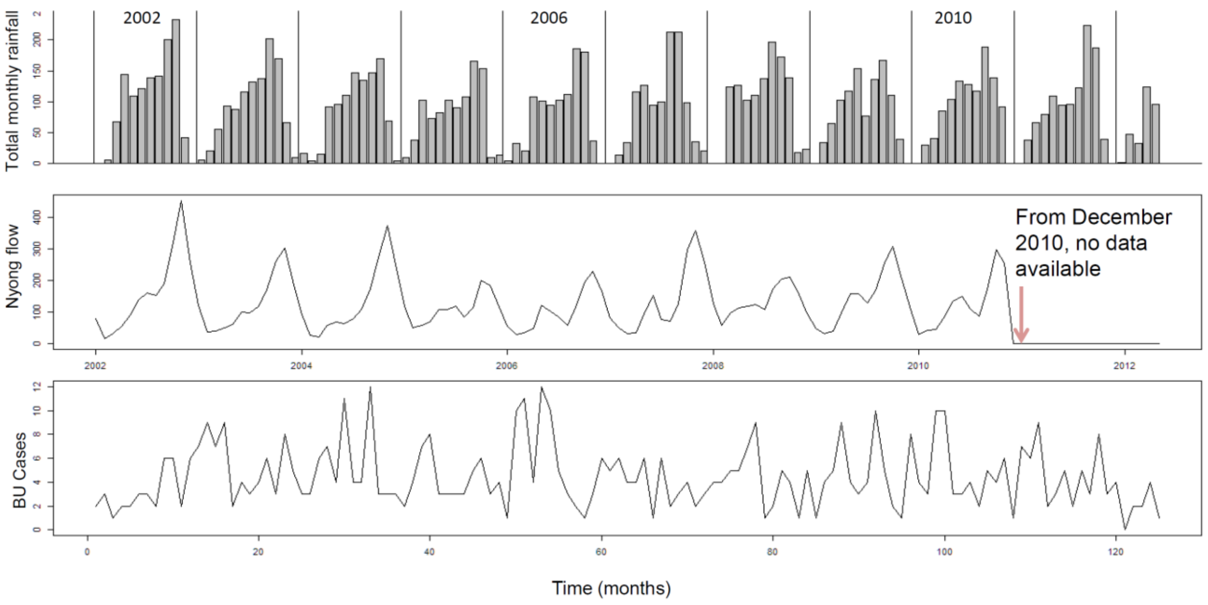

Technical Appendix Figure 4. Original time-series included in the analysis. From top to bottom: rainfall, Nyong River flow, and BU-cases time monthly series.

References

1. Landier J, Gaudart J, Carolan K, Lo Seen D, Guégan J-F, Eyangoh S, et al. Spatio-temporal dynamics and landscape-associated risk of Buruli ulcer in Akonolinga, Cameroon. PLoS Negl Trop Dis. 2014;8:e3123. PubMed http://dx.doi.org/10.1371/journal.pntd.0003123

2. Mensah-Quainoo E, Yeboah-Manu D, Asebi C, Patafuor F, Ofori-Adjei D, Junghanss T, et al. Diagnosis of Mycobacterium ulcerans infection (Buruli ulcer) at a treatment centre in Ghana: a retrospective analysis of laboratory results of clinically diagnosed cases. Trop Med Int Health. 2008;13:191–8. PubMed http://dx.doi.org/10.1111/j.1365-3156.2007.01990.x

3. World Health Organization. Buruli ulcer: diagnosis of Mycobacterium ulcerans disease. Portaels F, Johnson P, Meyers WM, editors. World Health Organization; 2001.

4. Pouillot R, Matias G, Wondje CM, Portaels F, Valin N, Ngos F, et al. Risk factors for buruli ulcer: a case control study in Cameroon. PLoS Negl Trop Dis. 2007;1:e101. PubMed http://dx.doi.org/10.1371/journal.pntd.0000101

5. Monitoring Agriculture with Remote Sensing [Internet]. 2013. Available from: http://www.marsop.info/

6. ORE BVET. BVET, Cameroon, Hydrological data. 2012.

7. Cazelles B, Chavez M, De Magny GC, Guégan J-F, Hales S. Time-dependent spectral analysis of epidemiological time-series with wavelets. J R Soc Interface. 2007;4:625–36. PubMed http://dx.doi.org/10.1098/rsif.2007.0212

8. Constantin de Magny G, Guégan J-F, Petit M, Cazelles B. Regional-scale climate-variability synchrony of cholera epidemics in West Africa. BMC Infect Dis. 2007;7:20. PubMed http://dx.doi.org/10.1186/1471-2334-7-20

9. Agier L, Deroubaix A, Martiny N, Yaka P, Djibo A, Broutin H. Seasonality of meningitis in Africa and climate forcing: aerosols stand out. J R Soc Interface. 2013;10:20120814. PubMed http://dx.doi.org/10.1098/rsif.2012.0814

10. Hashizume M, Chaves LF, Minakawa N. Indian Ocean Dipole drives malaria resurgence in East African highlands. Sci Rep. 2012;2:269. PubMed http://dx.doi.org/10.1038/srep00269
